# Barriers and enablers of adherence to infant nevirapine prophylaxis against HIV 1 transmission among 6-week-old HIV exposed infants: A prospective cohort study in Northern Uganda

**DOI:** 10.1371/journal.pone.0240529

**Published:** 2020-10-15

**Authors:** Agnes Napyo, Thorkild Tylleskär, David Mukunya, Josephine Tumuhamye, Milton W. Musaba, Anna Agnes Ojok Arach, Paul Waako, James K. Tumwine, Grace Ndeezi

**Affiliations:** 1 Department of Public Health, Faculty of Health Sciences, Busitema University, Mbale, Uganda; 2 Centre for International Health, University of Bergen, Bergen, Norway; 3 Department of Paediatrics and Child Health, Makerere University, Kampala, Uganda; 4 Department of Obstetrics and Gynaecology, Faculty of Health Sciences, Busitema University, Mbale, Uganda; 5 Department of Nursing, Lira University, Lira, Uganda; 6 Department of Pharmacology, Faculty of Health Sciences, Busitema University, Mbale, Uganda; Cornell University Joan and Sanford I Weill Medical College, UNITED STATES

## Abstract

**Background:**

Sub-optimal adherence to infant prophylaxis has been associated with mother-to-child-transmission of HIV. However, the factors associated have not been well characterised in different settings. This study describes barriers and enablers of adherence to infant prophylaxis among 6-week-old HIV exposed infants in Lira district, Northern Uganda.

**Methods:**

This prospective cohort study was conducted from 2018–2020 at the PMTCT clinic at Lira Regional Referral Hospital and included 472 mother-infant pairs. HIV-infected pregnant women were recruited, followed up at delivery and 6 weeks postpartum. We used a structured questionnaire to obtain data on socio-demographic, reproductive-related, HIV-related characteristics and adherence. Data were analysed using Stata to estimate adjusted risk ratios using Poisson regression models to ascertain barriers and enablers of adherence to infant nevirapine prophylaxis.

**Results:**

Barriers to infant adherence are maternal characteristics including: younger age (≤20 years adjusted risk ratio (ARR) = 1.55; 95% CI: 1.1–2.2), missing a viral load test during pregnancy (ARR: 1.4; 95% CI: 1.1–1.7) and not receiving nevirapine syrup for the baby after childbirth (ARR = 6.2; 95% CI: 5.1–7.6). Enablers were: having attained ≥14 years of schooling (ARR = 0.7; 95% CI: 0.5–0.9), taking a nevirapine-based regimen (ARR = 0.6; 95% CI: 0.4–0.9), long-term ART (≥ 60 months ARR = 0.75; 95% CI: 0.6–0.9), accompanied by a husband to hospital during labour and childbirth (ARR = 0.5; 95% CI: 0.4–0.7) and labour starting at night (ARR = 0.7; 95% CI: 0.6–0.8).

**Conclusion and recommendations:**

Despite mothers receiving nevirapine syrup from the health workers for the infant, non-adherence rates still prevail at 14.8%. The health system needs to consider giving HIV infected pregnant women the nevirapine syrup before birth to avoid delays and non-adherence. There is need to pay particular attention to younger women and those who recently started ART.

## Introduction

HIV-1 exposed infants (HEI) can get infected with HIV from their mothers during pregnancy, childbirth or breastfeeding. Over 90% of paediatric HIV infections are through mother-to-child transmission of HIV-1 (MTCT) [1]. However, giving antiretroviral therapy to the mother and infant prophylaxis to the infant during breastfeeding are the major interventions in the prevention of mother-to-child transmission of HIV-1 (PMTCT) [2, 3].

Since 2013, the World Health Organisation (WHO) consolidated guidelines on the use of antiretroviral drugs for treating and preventing HIV infection [3] advocate lifelong antiretroviral therapy (ART) regardless of immune status for pregnant and breastfeeding mothers in addition to infant prophylaxis for the baby for 6–12 weeks. A longer duration of prophylaxis is recommended for high-risk infants born to an HIV infected mother that has a viral load (VL) greater than 1000 copies/ml [2, 3]. For a high-risk infant, the mother’s VL test should be done at 12 weeks postpartum and only if < 1000 copies/ml should the infant stop taking nevirapine (NVP). If the maternal VL is not suppressed by 12 weeks, the infant should continue taking NVP until the mother’s VL is less than 1000 copies/ml or otherwise continue with NVP until four weeks after cessation of all breastfeeding [1]. These guidelines have been implemented in Uganda since 2012 [4]

For these interventions to yield impact in PMTCT, adequate adherence to both maternal ART and infant prophylaxis are a prerequisite [5]. Challenges in achieving optimal adherence can be programmatic, maternal- or infant-related. There are programmatic challenges with linkage of HEIs and their mothers from PMTCT to HIV care [6] and lack of clinic-based HIV counselling [7]. Maternal-related challenges include forgetfulness, poor adherence and social or cultural obligations [8]. Infant-related challenges are vomiting of the drug or the baby being sick [9]. Poor adherence to infant nevirapine prophylaxis may contribute to transmission of HIV hence identifying barriers to adherence is essential to eliminate MTCT.

Several studies have demonstrated an association between non-adherence to infant nevirapine prophylaxis and home deliveries, inadequate antenatal care, mother not receiving the nevirapine for her baby while at the hospital, misplacing of the baby’s drug, lack of transport and the mother staying with in-laws [10, 11].

While there are numerous benefits of ART prophylaxis for PMTCT, there are still disparities in rates of MTCT due to differences in programme settings, systems, support requirements and context. Varying ART adherence rates in various contexts can also contribute to the disparities in MTCT rates. Most studies done on adherence to infant nevirapine prophylaxis have been qualitative and were done under previous treatment paradigms and cannot be compared to today’s situation. Furthermore, different methods have been used to measure adherence to infant NVP prophylaxis like relying on caregivers’ report [12], measuring blood plasma concentrations of NVP therapeutic levels [9, 13] and electronic dose monitoring like medication event monitoring systems (MEMs) where bottle caps are fitted with a microchip that records the time and date of each bottle opening [14].

The goal set by the Joint United Nations Programme on HIV/AIDS (UNAIDS) of getting to zero new HIV infections among children is far from being achieved in Uganda [1] since Uganda’s MTCT rates of HIV have stagnated between 3–4% [15, 16] in the past decade. It is against this background that we studied barriers and enablers of adherence to infant nevirapine prophylaxis to optimise the PMTCT programme in Northern Uganda.

## Materials and methods

### Study design and setting

This prospective cohort study was conducted between August 2018 and January 2020 at the PMTCT clinic located within the Lira Regional Referral Hospital (LRRH). This clinic is an initiative of the Ugandan Ministry of Health where free HIV care and treatment are offered to HIV-infected pregnant women. These women have to attend several other clinics during pregnancy and after child birth such as: early infant diagnosis, postnatal, immunisation and family planning clinics. All these clinics are independent of each other and of the PMTCT clinic in terms of structural location. The PMTCT clinic receives about 30–50 HIV infected pregnant women daily. At this clinic, the women receive both their antenatal and routine HIV care until delivery. Approximately 600 HIV infected women deliver at LRRH annually. For delivery, women are free to choose any health facility or clinic. However, the nevirapine syrup for infant prophylaxis can only be provided at the clinic where the woman is registered for her HIV care. The reason for this is to assess, weigh and classify the baby as ‘high risk’ or ‘not high risk’ and to determine the dosage and duration of prophylaxis. It is rare for mothers to receive nevirapine syrup elsewhere. There are no stores of nevirapine syrup in the labour suite and maternity ward. Finally, when the baby is 6 weeks old, the mother-infant pair is transferred to the early infant diagnosis (EID) clinic for further management. Here, care may be extended, for instance with DNA PCR testing and viral load monitoring for the baby and others services as applicable.

### Participants and procedures

**Fig 1:**
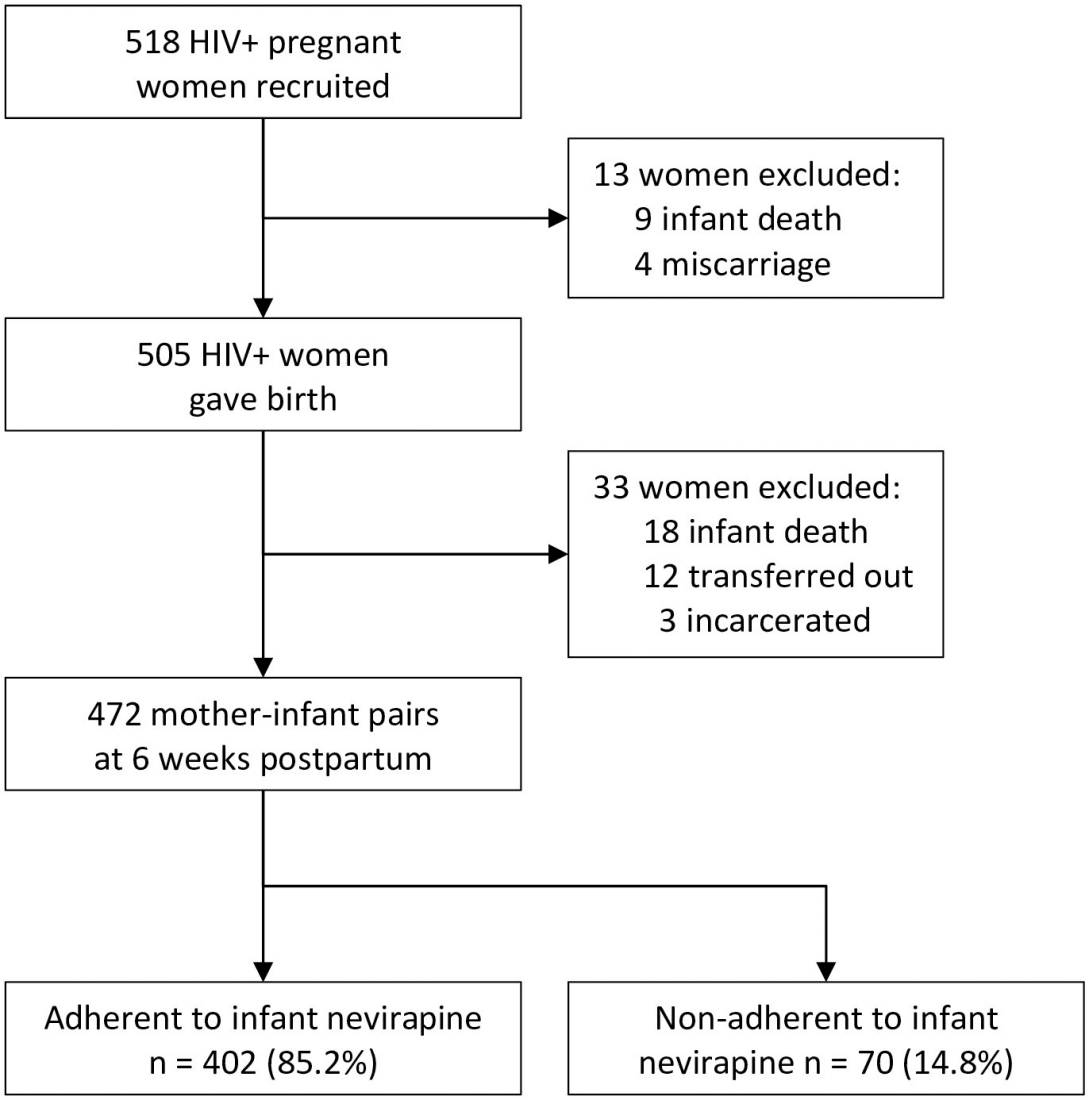
Study profile.

We consecutively enrolled HIV infected pregnant women who were receiving antenatal care at LRRH and with a gestational age of 20 weeks or more. After consent, women were interviewed on socio-demographic characteristics, HIV-related information like antiretroviral regimen, duration and a viral load test done during pregnancy. The interviews were conducted in *Lango* (the language predominantly spoken in the study setting) and/or English by trained study staff. The questionnaires were translated into *Lango* and back translated into English to minimize information and interpretation bias. The research assistants were trained, qualified midwives who had experience in conducting research, HIV counselling, providing antenatal care and taking off blood samples for viral load testing so as to shorten the waiting time of the mothers in the PMTCT clinic. To minimize loss to follow-up, information on telephone contacts and physical address were collected. The women were then followed up with a telephone interview around the time of delivery. At this point, women were interviewed on circumstances surrounding labour and delivery like time of onset of labour, type of delivery, place of delivery, person who supervised the delivery, maternal ART adherence, if the mother had received NVP syrup from the health worker at delivery and when the baby ingested the first prophylactic dose. At 6 weeks postpartum, mothers were followed up and asked about the infant’s adherence to nevirapine prophylaxis. A total of 472 mother-infant pairs were included in the final analysis, Fig 1. All study visits except the one around delivery were done at the PMTCT clinic as they coincided with the mothers’ routine visits for ART care. The 6-week interview was done just before the mother was transferred to the EID clinic. We, therefore, never got the chance to check for HIV transmission rates at 6 weeks from the DNA PCR results and hence this variable is not included in our analysis.

### Sample size estimation

We calculated a sample size for detecting an unknown proportion of infants adhering to infant prophylaxis using OpenEpi (openepi.com). We assumed a 50% proportion, 80% power, 95% confidence interval (CI) and 5% precision. The total sample size for this study was 384 HEI. After adjusting for 10% non-response and another 10% to allow for enough degrees of freedom in the multivariable analysis, the final sample size was 464. We included 472 mother-infant pairs.

### Variables

ART duration was recorded in months and any ART duration of ≥ 60 months (5 years) [17] was classified as long term ART, else as short term ART. Viral load test results during pregnancy were categorised as “<50 copies/ml”, “≥ 50 copies/ml” and “missing viral load”. Viral loads <50 copies/ml were referred to as ‘undetectable’ and those ≥50 copies/ml as ‘detectable’. The “missing viral load” was either due to having been on ART for less than 6 months or ineligibility for the annual viral load monitoring. Uganda ART guidelines do not include viral load monitoring at delivery. Women whose labour started between 06.00 hours to 18.59 hours were categorised as “day-time onset of labour” and else “night-time onset of labour”. Women who delivered in any type of health care setting were all categorised as “clinic delivery” and otherwise as “non-clinic delivery”. During the follow-up at the time of delivery, we also asked the mother if and when she had received the nevirapine syrup for the baby from a health worker and when the baby received the first dose. We estimated the expected date of delivery using the first day of the last normal menstrual period, and the research assistants called the mother at 7 days after the expected date of delivery. If the woman had not delivered yet, another telephone appointment was scheduled.

The outcome variable of interest was “non-adherence to infant nevirapine prophylaxis”. At the 6-week visit, we asked the mother, “In the past week, how many days did you miss to give the baby the nevirapine syrup?” Infants that had missed 0–2 doses of nevirapine syrup were collectively categorised and labelled as “adhered” and those that had missed three or more doses were collectively categorised and labelled “non-adherent” [9]. We relied on the mother’s recall for the measurement of infant adherence to NVP prophylaxis at 6 weeks postpartum [9, 14]. All infants included were brought to the PMTCT clinic by their mothers and so we never asked the mother if there is any other care giver that administered the nevirapine syrup to the baby other than the mother herself.

### Data analysis and management

Data was doubly entered into EpiData (www.epidata.dk, version 4.4.3.1) and exported for analysis to Stata version 14.0 (StataCorp, College Station, Texas, U.S.A.). Continuous data, if normally distributed, was summarised into means and standard deviations and if skewed, was summarised into medians with their corresponding interquartile ranges (IQR). Categorical variables were summarised into frequencies and percentages. The incidence of non-adherence to infant nevirapine prophylaxis was estimated and its confidence limits calculated using the exact method. Bivariable and multivariable analysis was done using Poisson regression models / analysis [18]. All variables that had a *p*-value < 0.25 at bivariable analysis and those of biological plausibility were collectively put into a multivariable model to control for confounding. We estimated unadjusted (RR) and adjusted risk ratios (ARR) with their corresponding 95% confidence intervals.

## Results

### Baseline characteristics

The mean age for the HIV positive pregnant women at baseline was 29.4 years (SD 5.4) (Table 1). Almost half of them were 30 years or more and had at least six years of schooling. The majority were unemployed and had disclosed their HIV status. More than half of them had taken ART for at least 5 years and had a viral load <50 copies/ml. All the women had someone accompanying them during labour and delivery, (Table 2). The majority had a spontaneous vaginal delivery in a hospital setting, were given nevirapine syrup by the health worker and were adherent to their ART.

**Table 1:** Baseline characteristics of the HIV infected pregnant woman at enrolment.

Maternal characteristics	Total N = 472 n (%)	Adherent to infant NVP prophylaxis N = 402 (85.2%) n (%)	Non-adherent to infant NVP prophylaxis N = 70 (14.2%) n (%)
**Age**			
≤ 20 years	28 (5.9)	20 (5)	8 (11.4)
21–29 years	206 (43.6)	171 (42.5)	35 (50)
30–39 years	225 (47.7)	198 (49.3)	27 (38.6)
≥ 40 years	13 (2.8)	13 (3.2)	0 (0)
**Education**			
≤ 6 years	233 (49.4)	195 (48.5)	38 (54.3)
7–13 years	167 (35.4)	141 (35.1)	26 (37.1)
≥ 14 years	72 (15.2)	66(16.4)	6 (8.6)
**Marital status**			
Married	441 (93.4)	382 (95)	59 (84.3)
Single	31 (6.6)	20 (5)	11 (15.7)
**Employment status**			
Employed	187 (39.6)	161 (40)	26 (37.1)
Unemployed	285 (60.4)	241 (60)	44 (62.9)
**Religion**			
Christian	454 (96.2)	386 (96)	68 (97)
Moslem	18 (3.8)	16(4)	2 (3)
**Ethnic group**			
Lango	430 (91.1)	367 (91.3)	63 (90)
Other	42 (8.9)	35 (8.7)	7 (10)
**Parity**			
0 to 4	337 (71.4)	289 (71.9)	48 (68.6)
5 to 9	135 (28.6)	113 (28.1)	22 (31.4)
**Gestational age**			
20–27 weeks	244 (51.7)	207 (51.5)	37 (52.9)
28–35 weeks	162 (34.3)	140 (34.8)	22 (31.4)
≥ 36 weeks	66 (14.0)	55 (13.7)	11 (15.7)
**HIV disclosure**			
Disclosed	457 (96.8)	392 (97.5)	65 (92.9)
Not disclosed	15 (3.2)	10 (2.5)	5 (7.1)
**Antiretroviral regimen**			
Efavirenz-based	423 (89.6)	356 (88.6)	67 (95.7)
Nevirapine-based	41 (8.7)	39 (9.7)	2 (2.9)
Protease inhibitor-based	8 (1.7)	7 (1.7)	1 (1.4)
**Antiretroviral treatment duration**			
Short-term (< 60 months)	298 (63.1)	243 (60.5)	55 (78.6)
Long-term (≥ 60 months)	174 (36.9)	159 (39.6)	15 (21.4)
**Viral load count**			
< 50 copies/ml	264 (56)	233 (58.1)	31 (44.3)
≥ 50 copies/ml	119 (25.3)	101 (25.2)	18 (25.7)
Missing viral load	88 (18.7)	67 (16.7)	21 (30)

**Table 2:** Maternal characteristics at delivery.

Characteristics	Total N = 472 n (%)	Adherent to infant NVP prophylaxis N = 402 (85.2%) n (%)	Non-adherent to infant NVP Prophylaxis N = 70 (14.8%) n (%)
**Onset of labour**			
Day time	251 (53.2)	210 (52.2)	41 (58.6)
Night time	221 (46.8)	192 (47.8)	29 (41.4)
**Attendant during delivery**			
Mother	89 (18.9)	72 (17.9)	17 (24.3)
Husband	109 (23.1)	102 (25.4)	7 (10)
Mother in law	76 (16.1)	61 (15.2)	15 (21.4)
Sibling	51 (10.8)	45 (11.2)	6 (8.6)
Other	147 (31.1)	122 (30.3)	25 (35.7)
**Type of delivery**			
Spontaneous vaginal delivery	413 (87.5)	350 (87.1)	63 (90)
Caesarean section	59 (12.5)	52 (12.9)	7 (10)
**Place of delivery**			
Clinic setting	441 (93.4)	379 (94.3)	62 (88.7)
Non-clinic setting	31 (6.6)	23 (5.7)	8 (11.4)
**Mother was given NVP syrup for baby at delivery**			
Given	362 (76.7)	341 (84.8)	21 (30)
Not given	110 (23.3)	61 (15.2)	49 (70)
**Maternal adherence to ART**			
Adhered	329 (69.7)	284 (70.7)	45 (64.3)
Did not adhere	143 (30.3)	118 (29.3)	25 (35.7)

### Non-adherence among infants at 6 weeks

Based on mothers’ recall, 402 of the infants (85.2%, 95% confidence interval (CI): 81.6%–88.3%) missed between zero and two doses of their nevirapine prophylaxis in the 7 days prior to the interview. A total of 70 infants (14.8% 95%CI: 11.7%–18.4%) missed between 3 and 7 doses in the week preceding the interview (Fig 1).

### Barriers and enablers of adherence to infant nevirapine prophylaxis

Barriers to adherence to infant nevirapine prophylaxis were the following maternal characteristics: younger age (≤20 years ARR = 1.55; 95% CI: 1.1–2.2), having missed to have a viral load test done during pregnancy (missing viral load ARR: 1.4; 95% CI: 1.1–1.7) and not receiving nevirapine syrup for the baby after childbirth (ARR = 6.2; 95% CI: 5.1–7.6). Maternal characteristics that enabled infant nevirapine adherence were maternal characteristics that include: having attained 14 or more years of schooling (ARR = 0.7; 95% CI: 0.5–0.9), taking a nevirapine-based regimen (ARR = 0.6; 95% CI: 0.4–0.9), having taken ART for a longer period of time (long-term (≥ 60 months) ARR = 0.75; 95% CI: 0.6–0.9), accompanied by her husband to hospital during labour and childbirth (husband ARR = 0.5; 95% CI: 0.4–0.7) and having labour start during the night-time (ARR = 0.7; 95% CI: 0.6–0.8) (Table 3).

**Table 3:** Barriers and enablers of adherence to infant to NVP prophylaxis among 6-week-old HIV exposed infants.

Variable	Unadjusted RR (95% CI)	Adjusted *RR (95%CI)
**Age**		
≤ 20 years	1.3 (1.1–1.9)	**1.5 (1.1–2.2)**
21–29 years	1	1
30–39 years	0.7 (0.6–0.9)	1.1 (0.9–1.4)
≥ 40 years	0.1 (0.01–0.5)	0.2 (0.03–1.3)
**Education**		
≤ 6 years	1	1
7–13 years	1.1 (0.9–1.3)	0.9 (0.8–1.1)
≥ 14 years	0.7 (0.5–0.9)	**0.7 (0.5–0.9)**
**HIV status disclosure**		
Disclosed	1	1
Not disclosed	2.1 (1.4–3)	1.3 (0.9–1.9)
**Antiretroviral regimen**		
Efavirenz-based	1	1
Nevirapine-based	0.4 (0.3–0.7)	**0.6 (0.4–0.9)**
Protease inhibitor-based	1.01 (0.4–1.9)	2.03 (0.98–4.2)
**ART duration**		
Short term (<60 months)	1	1
Long term (≥ 60 months)	0.6 (0.5–0.7)	**0.75 (0.6–0.9)**
**Viral load count**		
<50 copies/ml	1	1
≥ 50 copies/ml	1.2 (0.9–1.5)	1.1 (0.9–1.4)
Missing viral load	1.9 (1.5–2.3)	**1.4 (1.1–1.7)**
**Time of onset of labour**		
Day time	1	**1**
Night time	0.8 (0.7–0.97)	**0.7 (0.6–0.8)**
**Attendant during labour and delivery**		
Mother	1	1
Husband	0.5 (0.4–0.7)	**0.5 (0.4–0.7)**
Mother-in-law	0.8 (0.6–1.1)	0.8 (0.6–1.1)
Sibling	0.8 (0.6–1.1)	0.8 (0.6–1.1)
Other	0.9 (0.7–1.1)	0.9 (0.7–1.2)
**Mother was given NVP syrup for baby at delivery**		
Given	1	1
Not given	6.3 (5.2–7.6)	**6.2 (5.1–7.6)**
**Maternal adherence to ART**		
Adhered	1	1
Not adhered	1.3 (1.1–1.6)	1.1 (0.9–1.3)

*RR>1 refers to barriers, *RR<1 refers to facilitators.

## Discussion

In our study, we found that non-adherence to infant nevirapine prophylaxis was high, 14.8%. We relied on the mother or caregiver’s report in measuring adherence of the infant to nevirapine prophylaxis. Studies done in South Africa [9, 13, 19], have reported levels of non-adherence to infant nevirapine prophylaxis ranging from 12.3%–30% within which range the incidence of non-adherence in our study falls. The reported non-adherence rates vary across study contexts because different methods were used while measuring infant adherence. Studies that rely on self-reported or caregiver’s report [9, 10] have reported higher levels of non-adherence compared to those that have relied on electronic dose monitoring [9] and plasma concentration of therapeutic levels of nevirapine in the infant’s blood [9, 13]. All these studies measure adherence to infant nevirapine prophylaxis at different time points; some at birth and the majority at 6 weeks while factoring in variable recall periods. As much as this scenario could explain the disparities in rates of non-adherence across the different studies and ours, it is also likely that actual differences in adherence rates do exist in the different study contexts. The high incidence of non-adherence to infant prophylaxis in our study could be explained by the fact that many non-adherent women actually did not receive nevirapine for the baby after delivery.

For the purpose of this discussion we shall focus on barriers and enablers to infant nevirapine adherence that are important for policy. The barriers to infant adherence included younger maternal age, missed viral load test during pregnancy and mother not receiving infant’s nevirapine syrup after childbirth. Enablers to adherence included an HEI being born to a woman who: was well-educated (≥14 years of schooling), was taking a nevirapine-based regimen, has been on long-term ART, had a night-time onset of labour and was attended to by the husband during childbirth.

Our study showed that infants born to younger women were less likely to be adherent to their prophylactic treatment. A number of studies have shown similar findings [5, 9, 12]. Most young mothers in our cohort have taken ART for a shorter duration (less than 6 months). This means that their interface with the healthcare system is limited and that they have not had time to receive adequate ART adherence counselling and subsequently less informed about the necessity to adhere to treatment and prophylaxis. This is supported by the fact that babies born to women on long term ART in our cohort were likely to be more adherent to prophylaxis than those born to women on shorter duration of ART. This finding further demonstrates that women who have been on ART for longer periods are aware of the benefits of adherence to ART compared to their counterparts that have been on ART for shorter periods of time due to their frequent and routine interface with the health care system. One study [20] also demonstrated that women who have taken ART for shorter periods were more likely to report side effects of ART and this affected their adherence to ART. While conducting ART adherence counselling, health workers need to pay attention to these younger mothers.

In our study, infants born to women who did not receive nevirapine syrup from the health worker for the baby were likely not to adhere to infant ART prophylaxis. The majority of the women in our cohort who never received nevirapine for the baby actually had a non-clinic delivery, mostly a home delivery. Studies done in Zambia [13], South Africa [19] and a systematic review for sub-Saharan Africa [12] have demonstrated the association between home delivery and non-adherence to infant prophylaxis. Home deliveries have also been associated with the mother not receiving the nevirapine syrup for the baby from the health worker at the time of delivery [10, 12]. Women who have delivered at home may not be able to return to the hospital to pick the infant’s syrup for different reasons. Women who deliver outside the hospital are also less likely to receive counselling on the importance of their baby adhering to prophylaxis because they will deliver in the absence of a skilled birth attendant or health worker. Furthermore, HEIs born to women whose labour started in the night were more likely to adhere to nevirapine prophylaxis. Most women in our cohort are multiparous or of higher gravidity. The progress in labour for multiparous women is faster [14]. For women whose labour begins in the night are most likely to deliver during daytime which means they will be able to receive NVP syrup for the baby from the health worker at the PMTCT clinic. The PMTCT clinic is usually closed in the evening and night time in our study setting. Women who do not receive NVP for the baby are most likely not to administer it to the baby [12] and this contributes to non-adherence of the HEI to prophylaxis. An alternative strategy could be to provide all HIV infected pregnant women with NVP syrup for the baby from the ANC prior to delivery.

Infants born to women who had not had a viral load test done during pregnancy were less likely to adhere to the infant prophylaxis. The main reason for this was because they had been on ART for less than six months [2]. Having had less time in health care, these women have not yet benefitted from the on-going and continuous ART counselling. Women who have taken ART for shorter periods of time remain a critical target for adherence counselling.

In our study, it was shown that infants born to educated mothers were more likely to adhere to their prophylaxis. Educated mothers are more likely to read and comprehend concepts of adherence taught to them during ART adherence counselling sessions and therefore are more likely to support their infants with adherence to prophylaxis. Some studies have shown no association between maternal education and infant adherence to nevirapine prophylaxis [8] while other studies have shown an association between lack of maternal education and low infant nevirapine adherence [10, 12] just like our study.

Women who were taking an NVP-based regimen were more likely to have infants that adhere to infant prophylaxis. In our cohort, women taking NVP-based regimens have taken ART for longer periods of time. The benefits of taking ART for longer durations have already been discussed in the earlier paragraphs.

Women who were accompanied to the hospital by their husbands for labour and delivery were more likely to have infants that were adherent to their prophylaxis. The husband plays a key role in decision making when it comes to newborn care [21]. Male involvement in PMTCT generally improves adherence to the whole PMTCT programme. Other studies have actually demonstrated that male involvement in maternal and child health services promotes adherence to infant nevirapine prophylaxis [12, 22]. This finding evidently shows that if PMTCT programs in our study context and those similar to it promoted male involvement, this would not only enhance adherence to infant prophylaxis but to also the entire PMTCT cascade of interventions.

### Strengths and limitations

Most studies that have been conducted on this subject have been qualitative in nature. With this prospective cohort study we could explore these associations.

We relied on the mother’s or caregiver’s report for measuring adherence. Measuring medication adherence while relying on self-reporting varies is influenced by how questions are phrased and the period of recall. Our adherence estimates are likely to be over-estimated due to the recall bias and social desirability imposed by the self-reporting [13, 14]. However, other studies have demonstrated correspondence between relying on self-reports and other measures of adherence [9, 23].

Most studies have been conducted in urban settings. Our study was done in a rural context and our findings may only be generalizable to contexts similar to it. The definitions of adherence adopted, the methods used to measure it and recall periods varied across different studies. Therefore, comparing findings across these studies was rather difficult. We did not measure the infant’s adherence continuously from birth to six weeks of age considering the limitations of recall over such a long period of time. We also never included infant-related factors like infant refusal or illness that influence adherence to infant prophylaxis.

## Conclusion and recommendations

Despite many mothers receiving nevirapine syrup from the health workers for the infant, non-adherence rates still prevail. The barriers to adherence to infant NVP prophylaxis were in order of importance: mother not receiving nevirapine syrup for the baby after delivery, young maternal age and having missed to have a viral load test during pregnancy. The health system needs to consider to give HIV infected pregnant women the infant nevirapine syrup before birth to avoid delays and non-adherence. There is also a need to pay particular attention to younger women and those who recently started ART.

## Supporting information

S1 Data (XLSX)

## Abbreviations

ARR: Adjusted risk ratio
ART: Antiretroviral therapy
CI: confidence interval
HEI: HIV exposed infant
HIV: Human Immunodeficiency Virus
LRRH: Lira Regional Referral Hospital
MTCT: Mother-to-child transmission of HIV
NVP: Nevirapine
PMTCT: Prevention of mother-to-child transmission of HIV
